# On-Chip Quantitative Measurement of Mechanical Stresses During Cell Migration with Emulsion Droplets

**DOI:** 10.1038/srep29113

**Published:** 2016-07-04

**Authors:** D. Molino, S. Quignard, C. Gruget, F. Pincet, Y. Chen, M. Piel, J. Fattaccioli

**Affiliations:** 1 École Normale Supérieure - PSL Research University, Département de Chimie, 24 rue Lhomond, F-75005 Paris, France; 2 Sorbonne Universités, UPMC Univ. Paris 06, PASTEUR, F-75005, Paris, France; 3 CNRS, UMR 8640 PASTEUR, F-75005, Paris, France; 4 Laboratoire de Physique Statistique, Ecole Normale Supérieure, Université Pierre et Marie Curie, Université Paris Diderot, Centre National de la Recherche Scientifique UMR8550, 24 rue Lhomond, Paris 75005, France; 5 Institut Curie, CNRS UMR 144, 26 rue d’Ulm, 75005, Paris, France

## Abstract

The ability of immune cells to migrate within narrow and crowded spaces is a critical feature involved in various physiological processes from immune response to metastasis. Several *in-vitro* techniques have been developed so far to study the behaviour of migrating cells, the most recent being based on the fabrication of microchannels within which cells move. To address the question of the mechanical stress a cell is able to produce during the encounter of an obstacle while migrating, we developed a hybrid microchip made of parallel PDMS channels in which oil droplets are sparsely distributed and serve as deformable obstacles. We thus show that cells strongly deform droplets while passing them. Then, we show that the microdevice can be used to study the influence of drugs on migration at the population level. Finally, we describe a quantitative analysis method of the droplet deformation that allows measuring in real-time the mechanical stress exerted by a single cell. The method presented herein thus constitutes a powerful analytical tool for cell migration studies under confinement.

The ability of immune cells to migrate within narrow spaces is a critical feature involved in various physiological processes from immune response to metastasis. For instance, cells such as neutrophils are required to migrate within constrictions that are much smaller than their own diameter, such as small capillaries (*ca*. 2 μm)^1^, or extravasate to crowded environments such as inflamed tissues^2^. These abilities greatly depend on the mechanical properties of the cells, since, for example, an increase of the cell stiffness has been shown to increase their retention in blood capillaries, eventually leading to inflammation^3^,^4^.

The intracellular machine acting during cell migration is complex and involves cell surface adhesion molecules, cytoskeleton and its associated molecular motors, actin-plasma membrane interface, nucleoskeleton and mechano-transduction feedbacks^5^,^6^,^7^,^8^. *In vivo* assays of cell migration require the use of sophisticated microscopic techniques on live animals, such as *intravital microscopy*^9^, that are technically challenging. For the sake of simplicity and also to permit biophysical modeling of the migration processes, several *in-vitro* techniques have been developed^10^ as the modified Boyden chamber^11^ or transwell assay^12^ that provide end-point data but no information on cell behavior between the start and conclusion of the experiment.

Microfluidic technologies however, allow to quantitatively record in real-time the influence of the physical properties of the environment^13^ or the existence of spatiotemporal gradients^14^ on parameters such as migration speed^15^, directionality^16^,^17^,^18^,^19^,^20^ or polarity^21^. In confinement conditions, studies performed in microdevices have shown that nuclear deformability is one of the limiting factors that slows down and even impedes the ability of cells to migrate within microfabricated constrictions^22^,^23^,^24^,^25^.

From the materials point of view, the engineering of techniques relying on the analysis of deformable substrates such as thin silicon membranes^26^, 2D and 3D gels^27^,^28^,^29^ or flexible pillars^30^,^31^ largely improved our understanding about the stress generation pathways involved in cell migration. However the mechanical rigidity of the fabrication materials such as PDMS^32^ limits the collection of quantitative data related to the physical stress that a cell is able to produce when crossing a constriction during a migration event, thus pushing for the development of microdevices having softer actuation elements with mechanical properties comparable to those of cells^33^.

As an alternative to polymers or hydrogels that are more commonly used when soft substrates are needed^34^,^35^, we propose in this study to use oil-in-water emulsion droplets as *in vitro* mechanical sensors during cell migration, since their stiffness has been shown to be comparable to the one measured for cells^36^. Hence we developed a hybrid microchip made of parallel PDMS channels in which oil droplets, with sizes comparable to cells, are sparsely distributed and serve as deformable obstacles that migrating cells have to squeeze to explore their environment. Since the shape of a droplet is set by the interplay between the interfacial tension and the mechanical stress field acting on it^37^,^38^, a simple microscopic analysis of the deformation of the droplet shape over time brings quantitative information on the mechanical stress that cells are exerting on it.

After a description of the fabrication of the microdevice, we show that neutrophil-like HL-60 cells can cross and squeeze the obstacles while deforming their nucleus. We then describe the quantitative analysis procedure of the droplet deformation and we quantify the mechanical stress exerted by a cell on a droplet during crossing events. We finally show that the ability of a cell to pass a droplet obstacle is actomyosin dependent. Our system hence provides a simple *in vitro* tool to explore by live imaging the mechanic necessary for a cell to infiltrate narrow and crowded spaces as those present in tissues.

## Materials and Methods

### Emulsion droplets fabrication and staining

Oil droplets are made from soybean oil (Sigma-Aldrich, St. Louis, MO, USA). Briefly, soybean oil was dispersed and emulsified by hand in an aqueous continuous phase containing 15% w/w of Poloxamer 188 block polymer surfactant (CRODA, East Yorkshire, UK) and 1% w/w sodium alginate (Sigma-Aldrich, St. Louis, MO, USA) at a final oil fraction equal to 75%. The rough emulsion was sheared in a Couette cell apparatus at a controlled shear rate of 110 rpm as described by Mason *et al*.^39^. For storage and handling purposes the emulsion are diluted to an oil fraction of 60% w/w with 1% w/w of poloxamer 188 in the continuous phase and stored at 12 °C in a Peltier-cooled cabinet.

To stain droplets with Nile Red (Sigma-Aldrich, St. Louis, MO, USA), a red lipophilic dye, the droplets suspension is washed and resuspended in cell growth complete media containing 10 μM of Nile Red. Size distribution of the emulsion droplets was measured by brightfield microscopy and image analysis.

### Cell culture handling

HL-60 expressing GFP-Actin (kindly provided by Guillaume Charras, from UCL, UK) were grown in RPMI media supplemented with 15% fetal bovine serum, 50 mM Hepes, 2 mM L-Glutamine, 10 units/penicillin and 10 mg/ml streptomycin (Life Technologies, California, USA), in a 5% CO2-humidified atmosphere at 37 °C. Cells were passaged to 0.15 million cells per mL when a maximal density of 1–2 million cells per mL was reached (*ca*. every 2–3 days). Passages were done in a total volume of 10 mL pre-warmed culture medium, in 25 cm^2^ cell culture flasks with 0.2 μm filter cap (Nunc™, Roskilde, Denmark). HL60 cells were differentiated with 1.3% v/v DMSO for 6 days without antibiotics^40^. For all migration experiments cells were loaded in microchip in self-conditioned media.

### Cell staining and treatments

For nuclei staining cells where treated with 0.5 μg/ml Hoechst 33342 (Life Technologies, Carlsbad, California, USA) for 30 minutes at 37 °C. For fMLP treatment cells where treated with 100 nM, just before chip loading. For Y-27632 treatment, cells were loaded in chip and incubated for 4 h with 10 μM Y-27632, in self-conditioned media. For quantification of the Y-27632 effect only channels containing less than 4 drops where taken into account. In each channel the number of cells localized before the first droplet or after where counted along total distance of 700 μm from the loading well.

### PDMS microchips with cells and drops

The devices were made in PDMS (polydimethylsiloxane), using the standard soft lithography techniques^41^. In brief, we fabricated SU-8 (SU-8 3050, Microchem) masters on a silicon wafer, then proceeded to PDMS molding and thermal curing at 80 °C during two hours (RTV 615, 1:10 ratio for the reticulating agent, RTV 615, Momentive Performance Materials). PDMS chips were then glued to the coverslip of Fluorodish F35-100 (WPI, Sarasota, FL 34240, USA) as described by Vargas et *al.*^42^. After 30 min under vacuum, soybean drops pre-incubated in cell growth media were charged into loading wells at a 1:300 dilution. Cells were loaded immediately after at concentration of 10^5^ cells per well of 2,5 mm diameter. Cells were loaded in the microchip within their self-conditioned media, without any exogenous chemotactic agent.

### Microscopy

Live cells were imaged on a LSM 710 (Zeiss) confocal microscope, using a 405 nm laser diode exciting Hoechst 33342, a 488 nm argon laser line exciting GFP and a 561 nm diode laser line exciting Nile Red. Emission was detected between 410 and 480 nm for Hoechst 33342, 495–530 nm for GFP and 565–max nm for Nile Red. Live imaging studies were made at 37 °C in self-conditionned medium. Acquisition was made in channel-separated mode and with a line-scanning mode, with a line average of 2 and an 8-bit dynamic range. Images were analyzed using the softwares Fiji/Image J^43^ and Mathworks Matlab softwares.

### Interfacial tension measurement

To measure the interfacial tension of soybean oil droplets we used the micropipette-aspiration method that has been described in detail in a previous article^44^. Micropipettes were made from 1 mm borosilicate glass-tube capillaries (Harvard Apparatus, USA) that were pulled in a pipette puller (P-2000, Sutter instrument Co., USA) to tip diameters in the range of 2 to 3 μm. An oil hydraulic micromanipulator (Narishige, Japan) allowed for pipette positioning and micromanipulation. The pipette was connected to water reservoirs that could be translated vertically to apply precise suction pressures.

### Statistical analysis

Data were processed first with Wilcoxon test to evaluate data distributions. Data with Gaussian distributions were validated with paired t-test, non-Gaussian distributed sets of data were evaluated with Mann-Whitney. Graphs and statistics were obtained using GraphPad Prism software.

## Results

### Description of the hybrid microfluidic device

**Figure 1:**
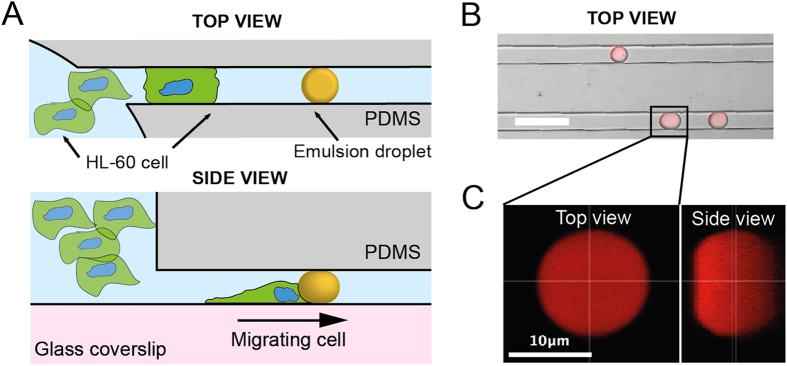
(**A**) Schematic view of the hybrid microdevice made from two circular loading chambers (diameter: 2.5 mm) connected by parallel rectangular channels (width: 14 μm, height: 8 μm). Soybean oil droplets are inserted within the channels and make a deformable obstacle to the migration of HL-60 cells. (**B**) Confocal microscopy picture of PDMS channels containing oil droplets stained with Nile Red dye. Scale bar: 30 μm. (**C**) Top view and 3D reconstructed side view of a droplet blocked in a micro channel. Scale bar: 10 μm.

We developed a microdevice (Fig. 1A) inspired by the micro-channel-based assays that have been used so far to study migration of immune cells under confinement^21^,^25^. The PDMS chip consists in two circular loading chambers (diameter: 2.5 mm) connected by 25 parallel channels with a rectangular cross-section (width: 14 μm, height: 8 μm, length: 2 mm). In each channel, soybean oil emulsion droplets having dimensions comparable to those of the channels are randomly distributed and serve as deformable obstacles on the path of migrating cells.

Emulsions are colloidal liquid-liquid metastable suspensions stabilized by a surfactant monolayer^45^ that have already shown their biocompatibility and their interest as liquid probes in a biophysical context^46^,^47^. To fabricate the droplets, we manually shear soybean oil in an aqueous solution of a polymeric surfactant (poloxamer 188) to stabilize the emulsion and a viscosifier (sodium alginate) to increase the viscoelasticity of the continuous phase and ease the fragmentation. The crude, polydisperse emulsion is then sheared in a Couette cell apparatus, following the method developed by Mason *et al*.^39^, to obtain a quasi-monodisperse, 13 ± 2 μm diameter emulsion (Figure S1A). Prior to the migration experiments, the continuous phase is replaced by normal cell culture medium after several centrifugation/rinsing steps.

After fabrication and mounting of the PDMS on a small Petri dish with a glass bottom, a dilute suspension of deformable soybean oil droplets is injected into one of the loading well at a concentration allowing the droplets to be sparsely distributed in the channels (Fig. 1B). The average diameter of the droplets was chosen to be slightly larger than the smallest dimension of the channels, so drops with a diameter bigger that 8 μm are squeezed horizontally and hence remain immobilized into the device. 3D reconstructions of Nile Red-stained droplets into the channels (Fig. 1C) show that droplets with a diameter larger than the channel width are pancake-shaped, the top and bottom interfaces being flat thanks to pressure exerted by the PDMS walls in the *z* dimension (Figure S2). We confirmed the shape of the droplets in this size range by numerical simulations (Figure S1B) made with the software Surface Evolver^48^.

### HL-60 migration in the microdevice

Cells of the immune system, such as HL-60, represent a simple model system to study cell migration without the need to derive cells from primary tissue^49^. The capability of differentiated HL-60 to bypass endothelial barriers renders this cell type particularly suitable for studying interstitial cell migration *in vitro*^50^,^51^,^52^. Moreover, this cell line has been extensively used as migrating cell model for experiments performed in microdevices^14^,^20^,^52^,^53^,^54^.

After insertion of the droplets in the microchannels, DMSO-differentiated^40^ HL-60 cells expressing GFP-actin are seeded in one of the loading well and the microchip is put at rest in a culture incubator to allow the cells to settle and recover their motility. After around 2 hours, cells spontaneously start entering channels where droplets are inserted, as observed by live imaging (Movie S1). HL-60 cells migrating within a microchannel move in the forward and backward direction relative to the loading well.

**Figure 2:**
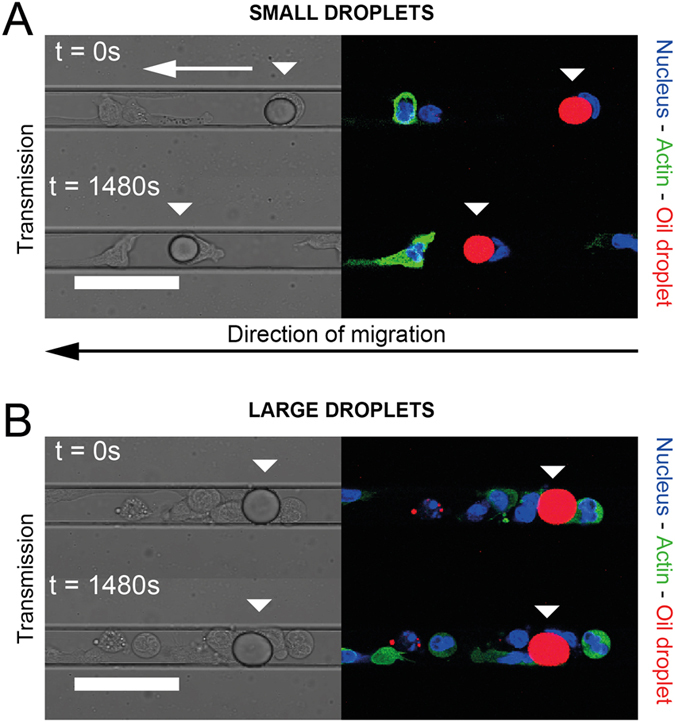
Time-lapse recording of the behavior of HL-60 cells (Hoechst 33342: blue, Actin-GFP: green) migrating along a channel and encountering a small (**A**) and a large (**B**) soybean oil droplet (Nile Red: red), the latter being large enough to be in contact with the walls of the PDMS microchannel. The relative positions of the arrows indicate the displacement of the droplets due to the cells. (**A**,**B**) Scale bars: 40 μm.

When a cell encounters a droplet within the microchannel, its behaviour towards the obstacle strongly depends on the size of the droplet. For droplets whose diameter is smaller than the width of the channel, HL-60 cells migrate while displacing the droplets with them over long distances, as shown in Fig. 2A and Movie S2. In the case of droplets that are as large as and larger than the microchannel width, cells are not able anymore to move the droplets while migrating but rather cross obstacle (Fig. 2B) and squeeze it sufficiently so the change of the droplet shape is easily observable with the microscope.

### Quantitative measurement of HL-60 invasion of the channels in presence of droplets

**Figure 3:**
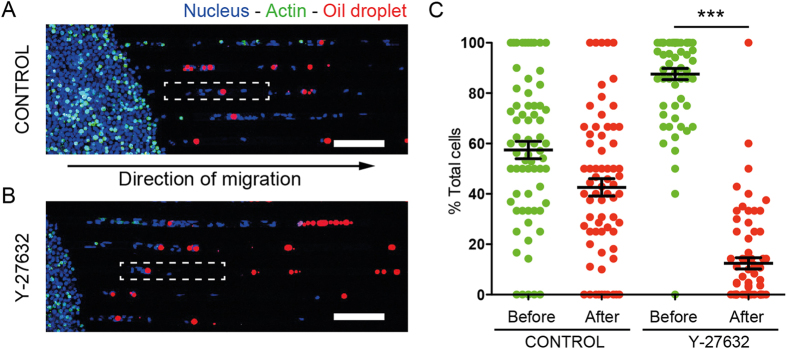
Comparison of the HL-60 spatial distribution along microchannels containing droplets in control and Y-27632 conditions. (**A**,**B**) Confocal microscopy mosaic images. Oil droplets are stained with Nile Red (red), cell nuclei with Hoechst 33342 (blue). Scale bars: 100 μm. (**C**). Percentage of cells localized before and after a droplet in a microchannel. N = 68 channels for a total of 3 independent experiments. For control P > 0.01 (t-test), for Y-27632 treatment, P < 0.0001 (Mann-Whitney).

We counted, for microchannels filled with sparsely distributed droplets, the number of cells localized either before or after the first droplet in a range of 700 μm distance from the loading well (Fig. 3A), in a case where cells are only loaded in one of the two inlets of the microchip. In addition to the control condition performed with the sole migration medium, and for the sake of comparison, we also performed the measurements in presence of Y-27632 that blocks ROCK1 kinase and in consequence myo-2 phosphorylation, necessary for actomyosin-dependent contraction^55^,^56^. We find that cells in control conditions are in average distributed equally before and after the droplets (Fig. 3B), whereas under Y-27632 condition, most of the cells remain localized in the region between the loading well and the first droplet of the micro channel. The drug treatment hence interferes with the capability of the cell to cross the droplets and invade the whole channel.

### Cells deform droplets during the crossing

**Figure 4:**
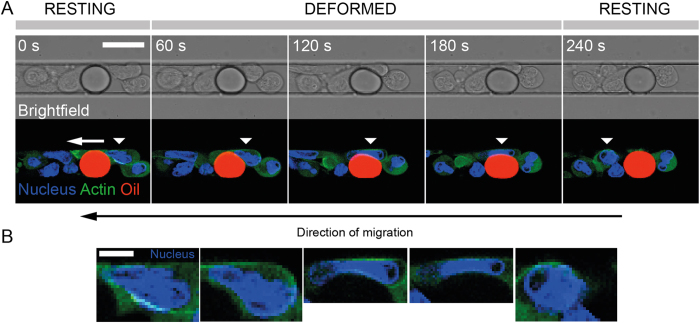
(**A**) Time-lapse recording of an HL-60 cell (Hoechst 33342: blue, Actin-GFP: green) migrating along a channel and squeezing a soybean oil droplet (Nile Red: red). The moving cell is identified with a white triangular mark. Scale bar: 15 μm. (**B**) Corresponding enlarged pictures of the nucleus of the migrating cell in (**A**). Scale bar: 5 μm.

Figure 4A shows a time-lapse recording of a crossing event recorded in the focal plane located in the middle of the microchannel. We see that both the cell and droplet are deformed during the encounter as a result of the mechanical stress applied by the cell on the droplet to go through it. While the nucleus of the neutrophil has a rounded aspect before and after the encounter with the droplet, the nucleus gets squeezed and elongates during the crossing. The maximal deformation of the droplet is observed when the cell nucleus is going from one side to the other of the droplet (Fig. 4B). The droplet, circular in its resting state, becomes pear-shaped when the cell is pushing on it, and finally recovers its resting shape when the cell moves away from it (Fig. 4A).

### Mechanical description of the droplet shape

The shape of an oil droplet at the equilibrium is governed locally by the interplay between its interfacial tension γ and the mechanical stress, homogeneous to a pressure, exerted by the environment on the droplet. As a consequence of the existence of an interfacial tension γ, according to the Young-Laplace equation^57^, the local shape of the interface of a droplet confined in a microchannel with an initial radius *R*_0_ obeys the general relationship, in spherical coordinates:


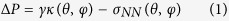


where 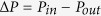 is the pressure excess across the emulsion interface, *κ*(*θ*, *φ*) the mean curvature of the interface and 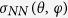 the normal mechanical stress acting on the droplet.

The mean curvature *κ*(*θ*, *φ*) writes as


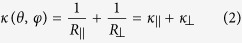


where 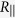 and *R*_⊥_ are the two principal radii of curvature.

In absence of hydrodynamic flow, *P*_*out*_ from Equation 2 is a constant equal to the hydrostatic pressure within the microchannel. Moreover, Fig. 4 and Figure S3 show that the HL-60 cells do not surround completely the droplet, meaning that a part of the oil interface is always in contact with the aqueous culture medium. As the inner pressure *P*_*in*_ is homogeneous within the droplet, its value is hence set by the oil/medium rather by the oil/cell membrane interfacial tension, which makes the excess pressure Δ*P* of the droplet a constant at each time point of the experiment.

The local mechanical stress 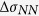 exerted by a cell can thus be measured according to the variation of the principal curvatures between the deformed and the resting state of the droplet within the contact area with the cell:


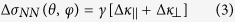


In its resting state, the principal radii of curvature are respectively measured in-plane (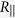) and out-of-plane (*R*_⊥_) to the focal plane of observation corresponding to the Fig. 4. In the following, we assume that the focal plane still remains a plane of principal curvature also in the case where the oil droplet gets deformed during the encounter.

Despite the large in-plane deformation of the droplet upon migration of a cell shown in Fig. 4, the out of plane deformation is almost inexistent (Figure S4), meaning that the corresponding curvature *κ*_⊥_ doesn’t change over time and its variation can be neglected in the mechanical stress computation.

A correct estimation of the stress 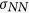 can hence be derived from the value of the interfacial tension and the in-plane curvature variation between resting and deformed droplet state:


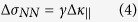


### Interfacial tension of the droplets

**Figure 5:**
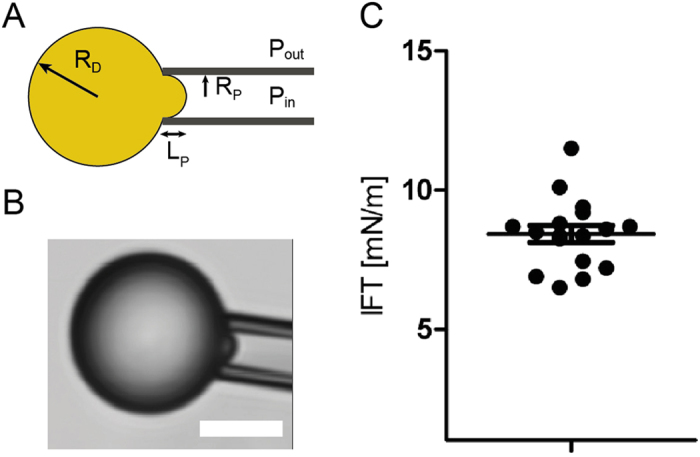
(**A**) Schematic view of the micropipette experiment used to measure the interfacial tension of the droplets. R_P_ and R_D_ are respectively the pipette and the droplet radius. (**B**) Bright field microscopy image of the droplets aspirated by the micropipette. Scale bar: 5 μm. (**C**) Plot showing the interfacial tension (IFT) of soybean oil droplets, dots are independent droplets (N = 17). Mean interfacial tension is 8.3 ± 1.26 mN.m^−1^.

To carefully evaluate the interfacial tension *γ* of the droplets suspended in the culture medium used for migration experiments, we used the micropipette aspiration method^44^. Upon aspiration by a very thin glass pipette, the droplet deforms and a spherical cap of radius R_c_ forms at the tip of the pipette (Fig. 5A). At equilibrium, the value of R_C_ depends on the interfacial tension γ of the droplets, the radius of the droplet R_D_, the aspiration pressure ΔP and can be expressed as:


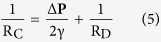


The aspiration ΔP corresponds to the pressure difference between the inside of the pipette and the external pressure. γ and R_D_ are constant throughout the experiment. Hence, varying ΔP induces changes in R_C_: the larger ΔP, the smaller R_C_. During the course of a measurement, ΔP is slowly increased and R_C_ decreases until it reaches the radius of the pipette R_P_. Up to that critical aspiration ΔP_c_, little change is observed in the geometry of the system. As soon as ΔP is larger than ΔP_c_, R_C_ becomes smaller than R_P_, which results in the sudden entry of the oil droplet in the pipette. This provides a direct measurement of the surface tension of the droplet:


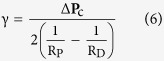


Each droplet can be blown out and aspirated again several times, allowing averaging the measurement and refining the interfacial tension value. The interfacial tension of the soybean oil droplets suspended into the differentiating media, at 37 °C, is equal to 8.4 ± 1.2 mN.m^−1^ (Fig. 5B).

### Segmentation and numerical computation of the in-plane curvature κ||

**Figure 6:**
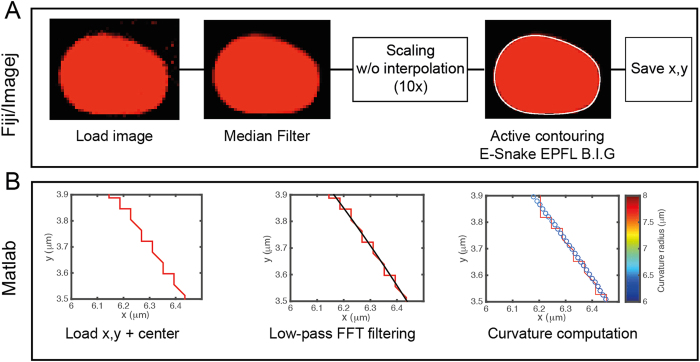
Workflow of the droplet segmentation routines. (**A**) Fiji/ImageJ segmentation procedure: confocal microscopy pictures of the droplets are first slightly denoised with a median filter then pictures are scaled 10 times and we used an active contour (*snakes*) routine to record the (x, y) Cartesian coordinates of the droplet interface after a visual comparison with the experimental picture. (**B**) Matlab curvature computation: the pixelation of the contour coordinates is suppressed by the application of a low-pass FFT filter. The local analytical curvature of the droplets shape is computed from the filtered data. Pictures show a zoomed area of the droplet contour.

To measure the curvature 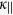 within the focal plane of observation, we proceed to a segmentation of the oil droplets and to their conversion in a shape that can be numerically processed to extract geometric data of interest (Fig. 6). Briefly, z stack images of the droplets stained with Nile Red were slightly denoised with a median filter to homogenize their aspect and ease the segmentation. Then, we used an active contour (*snakes*) routine to record the (x, y) Cartesian coordinates of the droplet interface. For the active contours to take a full account of the intensity gradient on the border of the droplets and ultimately increase the accuracy of the segmentation, the pictures were first scaled 10 times without interpolation to artificially subdivide each pixel of the original image in a 10 × 10 pixel grid (Fig. 6A). The pixelation of the contour coordinates was then suppressed by the application of a low-pass 1D FFT filtering both on the x and y coordinates, to get the interpolated model droplet. We finally computed the local analytical curvature of the droplets shape from the (x, y) Cartesian filtered data, as shown on (Fig. 6B).

### Mechanical stress exterted by the cells

**Figure 7:**
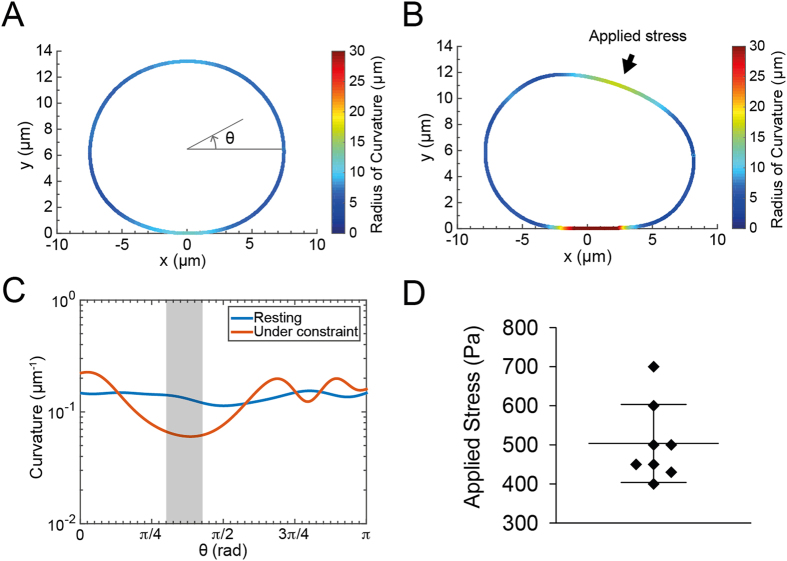
Resting (**A**) and constrained (**B**) droplet shape after segmentation and computation of the local radius of curvature. The shape outlines are color-encoded with respect to the local radius of curvature. (**C**) Analytical curvature as a function of the angular position along the droplet profile in the resting (blue) and constrained state (red). Only the values corresponding to the upper half part of the droplet are plotted. The grey area corresponds to the area where the mechanical stress applied by the cell is maximal. (**D**) Measured values of the mechanical stress exerted by the cells (N = 8).

From time-lapse recordings similar to the one shown in Fig. 4, we selected a frame where the droplet is resting, either before or after the crossing by a cell, and a frame where the droplets is squeezed and pear-shaped as a cell is pushing on it. Using the segmentation and computation routine described above, we obtain the shape outlines shown on Fig. 7A,B for resting and deformed oil droplet. For the sake of simplicity, the shape outlines are color-encoded with respect to the local radius of curvature. As expected when considering the simulations in Figure S1B, Fig. 7A shows that the droplet in the resting state is globally rounded and slightly flattened at locations where it touches the walls of the microchannel. When the cell pushes on the droplet to go though it, the radius of curvature increases in the region of contact with the cell and on the bottom part of the droplet. Figure 7C shows the evolution of the local analytical curvature along angular coordinates that correspond to the upper part of the droplet. The maximal mechanical stress is calculated at the location where the curvature of the constrained droplet is minimal (Fig. 7A,B - arrow). As shown on Fig. 7D, measurements performed on a sample of independent droplets (N = 8) give a numerical value 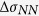 equal to 500 ± 100 Pa, which corresponds to a mechanical stress Δ*σ*_*NN*_ = 500 ± 100 pN.μm^−2^.

## Discussion

The microfluidic device described here allows monitoring migration and quantifying the ability of immune cells to migrate through deformable obstacles, both at population and at the single cell level. The chip design involves the trapping of quasi-monodisperse soybean oil-in-water droplets in PDMS microchannels within which DMSO-differentiated HL-60 cells migrate. Besides the HL-60 cell line, we also used primary murine dendritic cells (Figure S5), which successfully deformed droplets during their crossing, showing that both cell types, albeit different, can exert a mechanical stress on the obstacles.

When the droplets are large enough to be constricted by the four walls of the microchannel, they constitute immobile obstacles that migrating cells have to squeeze to go through. During this process, the cell nucleus gets strongly deformed and finally recovers its rounded shape when the cell reaches the other side of the obstacle. Although cells could potentially cross the droplets from one side to the other along any of the four walls of the microchannel, we observe that they actually never pass on the top and bottom flat sides of the pancake-shaped droplet but rather move along the parts where the droplets are rounded instead, which definitely ease the time-lapse observation (Movie S1). In a recent report, HL-60 cells have been shown to be sensitive to the local hydraulic pressure and hence use barotaxis to migrate preferentially in directions where the resistance is the lowest^54^. Although this property has been highlighted in a context where cells had to choose between two different channels with different hydrodynamic resistances, one can hypothesize that the same phenomenon is at work when a HL-60 chooses its local path to go through the droplet.

The choice of soybean oil as the formulation base was driven by the fact that it gives stable and biocompatible emulsions currently approved for pharmaceutical products^58^, but also because it has one of the lowest interfacial tension if we compare to common formulation constituents as silicone or mineral oils^59^. This insures that droplets are deformable enough to be squeezed by cells during the crossing, hence allowing the measurement of the change in the droplet shape over time. From the analysis of their deformation and the measurement of their interfacial tension, droplets are hence used as mechanical sensors during migration events, and the mechanical stress exerted by the cell on the droplet is computed from the local curvature change at the locations where the cell contacts the droplet.

In the expression of the Young-Laplace equation reported in Equation 2, we implicitly assumed that the value of interfacial tension *γ* is the same no matter if we consider the medium/emulsion interface or the part of the droplet in contact with the cell. This assumption, based on several previous reports from the literature^60^,^61^, is supported by the fact that a careful analysis of the droplet shape, e.g. on Fig. 4, doesn’t show any contact angle at the location where the cell membrane both is in contact with the droplet and the culture medium, which implies that the interfacial tension does not experience any noticeable change over the whole droplet shape.

To compute the mechanical stress exerted by the cells from the analysis of the droplet shape, we neglected in Equation 3 the variation of the out-of-plane curvature 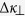, considering the *z*-stack pictures shown on Figure S4. Whereas the *xy* imaging resolution is sharp enough to measure precisely the in-plane curvature 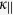 within the focal plane, the necessity to acquire confocal images on a large area, with 4 color channels and with a reasonable temporal dynamics forbade us to acquire more that 8 stacks per droplet in the *z* direction, thus making difficult to numerically quantify the curvature evolution between the two pictures of Figure S4. Despite the sole existence of a qualitative visualization, the assumption that the change in the out-of-plane curvature is negligible during the crossing event is supported by the geometry of the channels, whose height is almost two times smaller than their width, thus making more difficult for a cell to deform the interface in the *z* direction than the one in the focal plane.

The mechanical stress we measure from the analysis of the oil droplet deformation (Δ*σ*_*NN*_ = 500 ± 100 pN.μm^−2^) is in the middle range of what has been measured so far for traction stresses of migrating cells, both in 2D and 3D conditions^27^,^62^,^63^,^64^,^65^. Several studies have shown that the organelle that limits the most cell deformation during migration in constrictions is the nucleus^3^,^66^,^67^, since it is the biggest and stiffest organelle of the cell^67^. From Fig. 4B, we can estimate the smallest dimension the nucleus can reach when fully deformed is around 2 microns, which is in accordance with data from the literature^22^,^24^. The mechanical stress value we provide here is thus related to the ability of a cell to deform an inert object of comparable stiffness in order to cross it, and can be seen as the amount of force the cell has to provide to create a space large enough so that its nucleus can go from one side of the droplet to the other.

Knowing the value of the mechanical stress exterted by the cells, we can now give an *a posteriori* justification of the advantage to use oil droplets as actuation element instead of solid materials as polymers or hydrogels. Indeed, replacing the droplet with a more common elastic material would imply to work in the lower end of the possible Young’s moduli^35^ (*ca.* 1 kPa). Although such materials have been widely used for mechanosensitivity studies, in our case they would be hardly processable, from the microfabrication point-of-view, at the scale we are interested in.

In presence of Y-27632, a drug that inhibit the actomyosin contractility, our results show that differentiated HL-60 remain motile, which is in accordance with former measurements performed in absence of exogenous chemotactic agent^50^. However, cells are not able anymore to cross the droplets (Movie S3), and they stay stuck on one side of the obstacles instead. This shows that the mechanical stress we measure rely on actomyosin contractility, the physiological mechanism that cells use to squeeze the obstacle and move on. Whether or not the mechanical stress that cells exert on the droplets depends on the rigidity of the obstacle or the nucleus deformability are questions that our system make possible to address.

In conclusion, we believe that, as compared to common migration assays that have been developed so far and that have been summarized earlier in this report (Boyden chamber, transmigration assay, microchannel and confinement, etc.), our method increases the dimensionality of the cell migration analysis: in addition to the measurement of physical parameters such as speed or directionality, using droplets as sensors allows measuring the mechanical stress that cells exert while migrating, along to probing the invasiveness of immune cells in a crowded confinement, and exploring by fluorescent microscopy the molecular machinery necessary for generating such stress. Moreover, the possibility to functionalize the interface of the droplets with adhesive molecules or proteins^46^,^68^ would make possible to decipher the interplay between adhesion on the obstacles and migration.

## Conclusion

In this work, we developed a novel type of hybrid microchip that allows monitoring cell migration in real time and quantifying the mechanical stress they exert while migrating in crowded and narrow channels. The microchip is made of a set of parallel PDMS channels with a rectangular cross-section in which oil droplets are sparsely distributed and serve as deformable obstacles cells have to squeeze to explore their environment. This system is easy to handle and a simple microscopic analysis of the deformation of the droplet shape over time brings quantitative information on the mechanical stress that cells are exerting on it, and allows exploring by fluorescent microscopy the intracellular and biochemical events associated to this process.

## Additional Information

**How to cite this article**: Molino, D. *et al*. On-Chip Quantitative Measurement of Mechanical Stresses During Cell Migration with Emulsion Droplets. *Sci. Rep.* **6**, 29113; doi: 10.1038/srep29113 (2016).

## Supplementary Material

Supplementary Information

Supplementary Movie S1

Supplementary Movie S2

Supplementary Movie S3
