# Supplementary material for: On-Chip Quantitative Measurement of Mechanical Stresses During Cell Migration with Emulsion Droplets
Source: Sci Rep. 2016 Jul 4;6:29113. doi: 10.1038/srep29113 (PMC4931467; doi:10.1038/srep29113)
Supplement: Supplementary Information [file srep29113-s1.pdf]

ON-CHIP QUANTITATIVE MEASUREMENT OF MECHANICAL STRESSES DURING CELL MIGRATION WITH EMULSION DROPLETS

D. Molino[1,2,3], S. Quignard[1,2,3], C. Gruget[4], F. Pincet[4], Y. Chen[1,2,3], M. Piel[5], J. Fattaccioli[1,2,3]

SUPPLEMENTARY INFORMATIONS

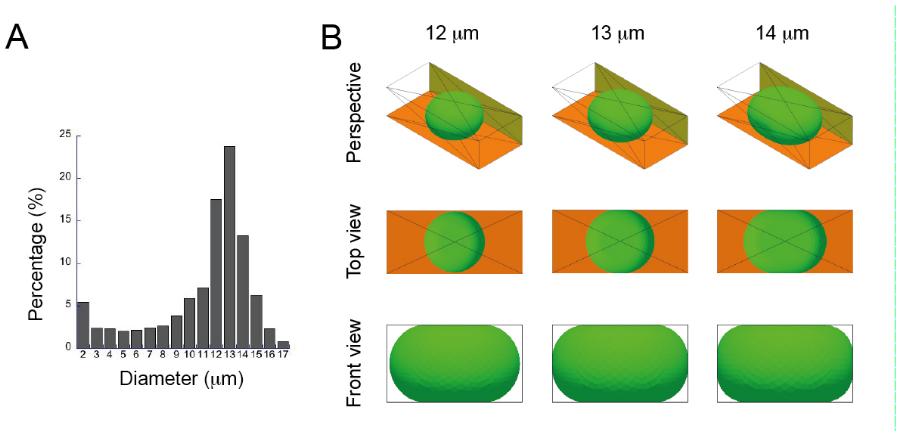

Figure S 1: (A) Size distribution of soybean oil droplets preparation measured from microscopy images. The size distribution was measured by transmission microscopy and image analysis using NIH Image J. (B) Simulation of the shape of a droplet confined in a microchannel (height = 8 μm, width = 14 μm) as a function of its initial diameter (Surface Evolver [1]).

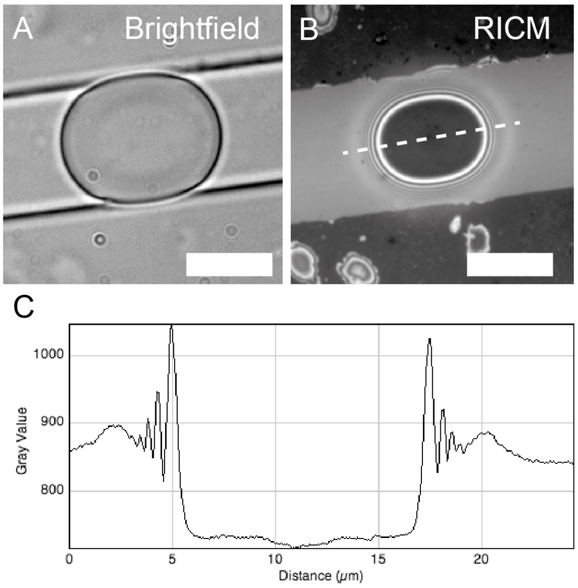

Figure S 2: (A) Brightfield image of a soybean oil droplet in a microchannel (equatorial plane). (B) RICM image of the contact area between the droplet and the bottom glass coverlip of the microchip. (C) Intensity profile along the dashed line on the RICM picture. The intensity is constant within the contact area. Scale bar: 10 μm.

Reflection Interference Contrast Microscopy. RICM, originally developed by I. Curtis [2] to image the focal adhesion of cells attached on glass, is a microscopy technique that uses interferences of light reflected from closely apposed surfaces, here the glass coverslip and the oil/water interface, to provide an image containing information about the separation of those surfaces[3]. In our experiments, RICM images were acquired using a Cy3 fluorescence filter set to which we removed the emission filter so a green monochromatic and non-coherent light could be used for the epi-illumination and image recording. The interference fringes give spatial information about the profile of the droplet in close vicinity with the glass coverslip.

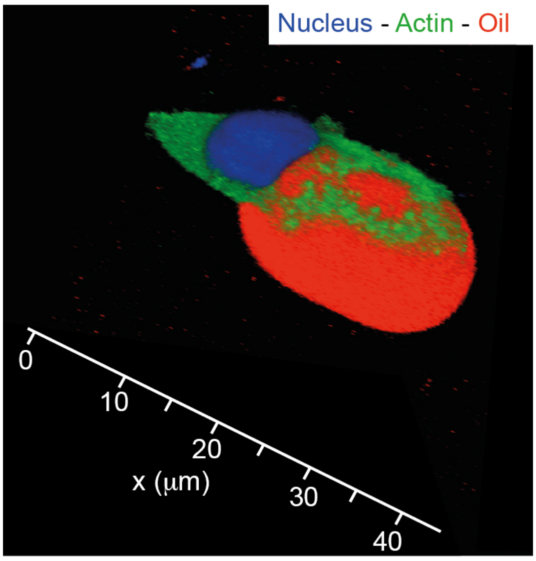

Figure S 3: 3D reconstructed perspective view of a migrating HL-60 cell (blue: nucleus, green: actin) squeezing a soybean oil droplet in a micro channel (red).

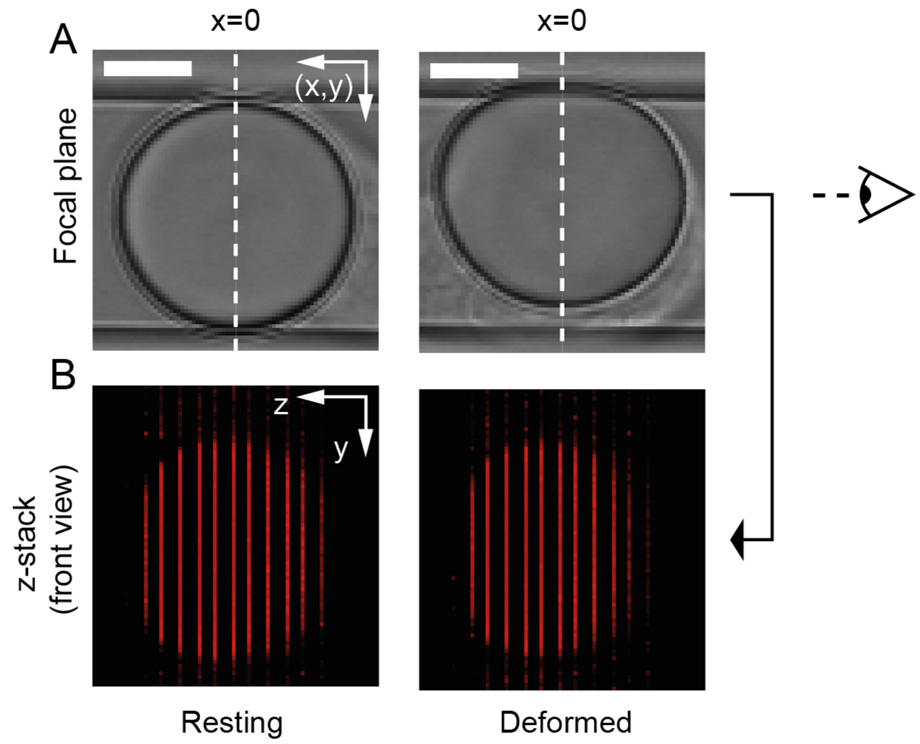

Figure S 4: (A) Bright field top view of resting and deformed droplet within a micro channel. (B) Confocal z-stack front view of the droplet profile along the vertical plane (x=0) depicted by the dashed line in (A). Scale bar: 5 μm.

Estimation of the out-of-plane curvature variation $\Delta\kappa_{\perp}$. Time-lapse recordings are performed in 5D conditions (color, time, space) with a confocal microscope. For a droplet in a microchannel, 8 frames spaced by 1 micron are acquired in the *z*-direction to record maximum information on the droplet deformation.

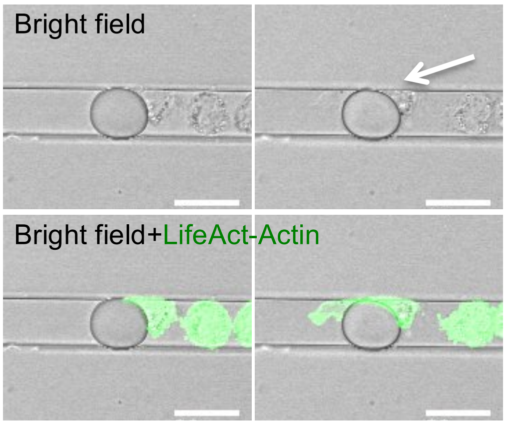

Figure S 5: Primary dendritic cells from wild type mice expressing LifeAct-GFP (actin in green) migrating in a micro channel with a blocked oil droplet. Scale bar: 15µm.

Dendritic cells culture handling. Mouse bone-marrow derived dendritic cells (BMDCs) were cultured during 10-12 days in IMDM medium containing 10% Calf serum, 20mM Glutamine, 100U/ml Pen-Strep, 50µM 2-βMe and granulocyte–macrophage colony-stimulating factor-containing supernatant obtained from transfected J558 cells, as previously described [4].

Movie S1: Time-lapse recording of HL-60 cells migrating in a PDMS micro-channel blocked with an emulsion droplet (red : droplet, green : actin, blue : nucleus). Scale bar: 40 μm.

Movie S2: Comparison of the behaviour of HL-60 cells encountering a small (top) and large (bottom) droplet within the micro-channel (red : droplet, green : actin, blue : nucleus). Scale bar: 40 μm

Movie S3: Time-lapse recording of Y-27632-treated HL-60 cells encountering a droplet (blue : nucleus). Scale bar : 20 μm.

REFERENCES

1. Brakke, K. A. The Surface Evolver. *Exp. Math.* **1,** 141–165 (1992).

2. Curtis, A. S. G. A Study by Interference Reflection Microscopy. *J. Cell Biol.* **20,** 199–215 (1964).

3. Rädler, J. & Sackmann, E. Imaging optical thicknesses and separation distances of phospholipid vesicles at solid surfaces. *J. Phys. II* **3,** 727–748 (1993).

4. Faure-André, G. *et al.* Regulation of dendritic cell migration by CD74, the MHC class II-associated invariant chain. *Science* **322,** 1705–10 (2008).
